# Dietary supplementation with probiotics during late pregnancy: outcome on vaginal microbiota and cytokine secretion

**DOI:** 10.1186/1471-2180-12-236

**Published:** 2012-10-18

**Authors:** Beatrice Vitali, Federica Cruciani, Maria Elisabetta Baldassarre, Teresa Capursi, Enzo Spisni, Maria Chiara Valerii, Marco Candela, Silvia Turroni, Patrizia Brigidi

**Affiliations:** 1 Department of Pharmaceutical Sciences, University of Bologna, Bologna, Italy; 2 Department of Gynecology, Obstetrics and Neonatology, University of Bari, Bari, Italy; 3 Department of Experimental Biology, University of Bologna, Bologna, Italy

## Abstract

**Background:**

The vaginal microbiota of healthy women consists of a wide variety of anaerobic and aerobic bacterial genera and species dominated by the genus *Lactobacillus*. The activity of lactobacilli helps to maintain the natural healthy balance of the vaginal microbiota. This role is particularly important during pregnancy because vaginal dismicrobism is one of the most important mechanisms for preterm birth and perinatal complications. In the present study, we characterized the impact of a dietary supplementation with the probiotic VSL#3, a mixture of *Lactobacillus*, *Bifidobacterium* and *Streptococcus* strains, on the vaginal microbiota and immunological profiles of healthy women during late pregnancy.

**Results:**

An association between the oral intake of the probiotic VSL#3 and changes in the composition of the vaginal microbiota of pregnant women was revealed by PCR-DGGE population profiling. Despite no significant changes were found in the amounts of the principal vaginal bacterial populations in women administered with VSL#3, qPCR results suggested a potential role of the probiotic product in counteracting the decrease of *Bifidobacterium* and the increase of *Atopobium*, that occurred in control women during late pregnancy. The modulation of the vaginal microbiota was associated with significant changes in some vaginal cytokines. In particular, the decrease of the anti-inflammatory cytokines IL-4 and IL-10 was observed only in control women but not in women supplemented with VSL#3. In addition, the probiotic consumption induced the decrease of the pro-inflammatory chemokine Eotaxin, suggesting a potential anti-inflammatory effect on the vaginal immunity.

**Conclusion:**

Dietary supplementation with the probiotic VSL#3 during the last trimester of pregnancy was associated to a modulation of the vaginal microbiota and cytokine secretion, with potential implications in preventing preterm birth.

**Trial registration:**

ClinicalTrials.gov NCT01367470

## Background

The vaginal microbiota of healthy women consists of a wide variety of anaerobic and aerobic bacterial genera and species dominated by the facultative, microaerophilic anaerobic genus *Lactobacillus*[1]. The activity of lactobacilli helps to maintain the natural healthy balance of the vaginal microbiota. This role is particularly important during pregnancy because abnormalities in vaginal communities, such as bacterial vaginosis (BV) and aerobic vaginitis (AV), have been claimed as important mechanisms responsible for preterm birth and perinatal complications [2].

The association of lower genital tract infection with an increased risk of preterm delivery and preterm rupture of the fetal membranes has recently attracted great interest in the pathogenesis of such infection-related mechanisms [3,4]. Earlier studies showed an increased rate of prematurity in women with BV, an alteration of the endogenous vaginal microbiota associated with decreased levels of hydrogen peroxide-producing *Lactobacillus* species [4-6]. The mechanisms linking BV with preterm delivery have not been fully identified, but local immune response is hypothesized to be crucial. Despite the notion that BV is a non-inflammatory condition, evidence exists that demonstrates altered levels of certain pro-inflammatory cytokines in women with BV [7,8].

Parturition is characterized by cervical ripening and myometrial maturation with subsequent uterine contractions leading to cervical dilatation and birth [9]. The process of labor displays many of the hallmarks of inflammation. Acute inflammatory features, such as increased influx of leucocytes and elevated expression of pro-inflammatory cytokines, have been observed in cervical tissues and fetal membranes during both term and preterm labor [10-12].

A potentially novel way to protect against infection-mediated preterm birth is to use probiotic bacteria, especially lactobacilli. Probiotics, defined as “live microorganisms which, when administered in adequate amounts, confer a health benefit on the host” [13], are being studied for their ability to replenish vaginal lactobacilli and modulate immunity [14-16]. In addition, administration of probiotics to the mother during pregnancy and breast-feeding has been described by some studies as a safe and effective mode of enhancing the immunoprotective potential of the breast milk and preventing atopic eczema in the infant [17,18].

In recent years, culture-independent techniques based on the analysis of rRNA gene sequences have been developed, providing powerful tools to reveal the phylogenetic diversity of the microorganisms found within vaginal microbiota and to understand community dynamics [19-24]. In particular, PCR-denaturing gradient gel electrophoresis (PCR-DGGE) has been successfully used to identify the bacterial composition of different ecological niches, including the vaginal microbiota [22,25,26]. Real-time PCR is a powerful technique for the quantitative analysis of specific microbial populations belonging to complex ecosystems [22,27,28]. Specific primers can be used to focus the quantitative analysis on a particular genus, species or strain of interest.

Several bacterial species are known to colonize both the gastrointestinal and the reproductive tract, and the rectum has been suggested to play an important role as a source or reservoir for organisms that colonize the vagina [15,29]. On this basis, the aim of the present study was to evaluate the impact of a dietary supplementation with the probiotic product VSL#3, a mixture of *Lactobacillus*, *Bifidobacterium* and *Streptococcus* strains, on the vaginal microbiota and immunological profiles of asymptomatic healthy women during late pregnancy. The dynamics of the vaginal bacterial communities prior and after probiotic ingestion were assessed by PCR-DGGE and real-time PCR, while the modulation of the cytokine secretion in vaginal fluids was measured by Luminex® Immunoassay. Although previous studies demonstrated the therapeutic efficacy of VSL#3 in the management of gastrointestinal disorders, especially inflammatory bowel disease [30], as well as the ability of the VSL#3 strains to colonize the gut environment [31] and to modulate the immune response of the colonic mucosa [32], this is the first study that investigates the indirect effects of this probiotic formula on the vaginal microbiota.

## Results

### Bacterial population profiling with PCR-DGGE

**Figure 1:**
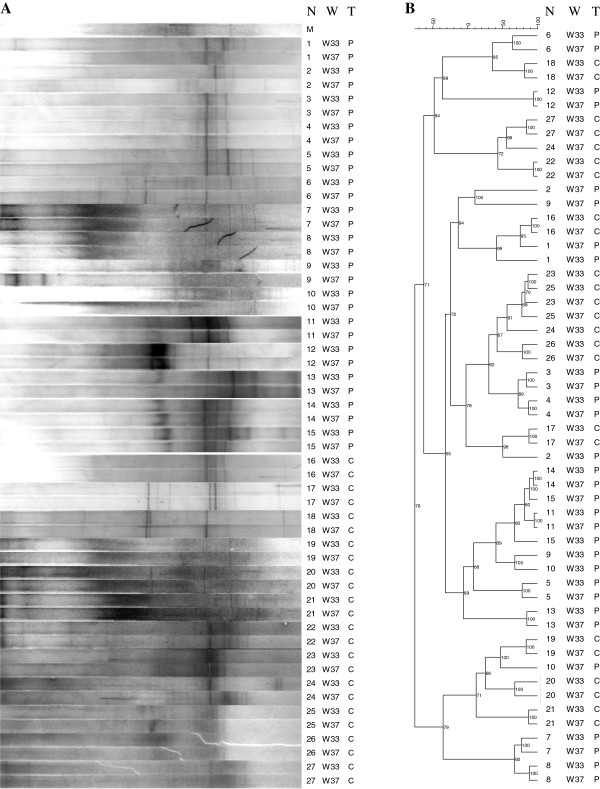
**PCR**-**DGGE analysis with universal primers for bacteria.** Analysis was conducted on the vaginal samples collected at 33^rd^ (W33) and 37^th^ (W37) week of gestation from 15 women supplemented with the probiotic VSL#3 [(P) N. 1–15] and 12 control women [(C) N. 16–27]. N: woman number; W: week of gestation; T: type of supplementation. (**A**) PCR-DGGE fingerprints. M, external reference marker. (**B**) Dendrogram of the DGGE profiles shown in panel **A**. Pearson correlation was used to calculate the similarity in DGGE profiles.

PCR-DGGE analysis with universal primers for bacteria (HDA1-GC/HDA2) was used to investigate: (i) the stability of the predominant vaginal bacterial communities over a period of 4 weeks in the last trimester of pregnancy, from the 33^rd^ (W33) to the 37^th^ (W37) week of gestation, and (ii) the influence of the oral consumption of the probiotic VSL#3 from W33 to W37 on the predominant vaginal microbiota (Figure 1).

DGGE band profiles displayed a relatively low complexity for both probiotic (P) and control (C) groups, as assessed by the richness index. Mean values of the richness index were 6.6 at both W33 and W37 for C group and shifted from 8.4 (W33) to 7.4 (W37) for P group without significant variations between W33 and W37.

Pearson correlation was used to calculate the similarity index (SI) between DGGE patterns related to the time points W33 and W37 for each pregnant woman (Table 1). The SI median values of P group and C group were 73% and 79%, respectively. In particular, 3 women belonging to P group (N. 2, 9 and 10) and only one woman belonging to C group (N. 24) showed SI values lower that 50%. For each woman, significant differences between DGGE profiles related to W33 and W37 were searched by Wilcoxon Signed Rank Test. No significant variations were detected between W33 and W37 in control women. Significant differences (*P* < 0.05) were found for 5/15 (33%) women belonging to P group (N. 4, 5, 9, 10, 11). Interestingly, women N. 9 and 10 were the same presenting SIs < 50%. These data suggested a potential role of the probiotic formula in modulating the vaginal bacterial communities. The peak heights of the DGGE densitometric curves were analyzed using the Wilcoxon Signed Rank Test in order to search for significant differences in single species abundances between W33 and W37. No significant changes in species abundance were found for both P and C groups, even in women N. 4, 5, 9, 10, 11.

**Table 1:** **Similarity index** (**SI**) **of DGGE profiles related to W33 and W37 obtained with universal** (**HDA1**/**HDA2**) **and *Lactobacillus***-**specific** (**Lac1**/**Lac2**) **primers**

**Woman N**	**HDA1**-**GC**/**HDA2 SI (%)**	**Lac1**/**Lac2**-**GC SI (%)**
Probiotic (P)		
1	55.2	21.6
2	28.4	62.0
3	84.0	84.0
4	87.7	84.1
5	78.0	87.8
6	64.5	68.1
7	77.2	85.6
8	88.5	95.5
9	37.5	86.2
10	41.3	91.9
11	95.3	96.6
12	94.5	93.3
13	84.7	96.9
14	94.3	94.3
15	81.1	44.5
Control (C)		
16	91.2	90.9
17	87.8	93.7
18	81.6	76.9
19	83.7	91.5
20	67.7	81.3
21	87.1	94.3
22	94.6	74.4
23	85.3	74.1
24	25.4	46.0
25	84.7	84.2
26	78.3	68.1
27	84.5	86.3

Cluster analysis showed that the DGGE profiles related to the time points W33 and W37 clustered together for all the control women, except for the woman N. 24 (Figure 1). Four supplemented women (N. 2, 9, 10 and 15) showed W33 and W37 DGGE profiles not closely related. However, the DGGE patterns of the majority of the women administered with VSL#3 grouped according to the subject and not to the time point, revealing that the inter-individual variability was higher than the variability induced by the probiotic supplementation.

**Figure 2:**
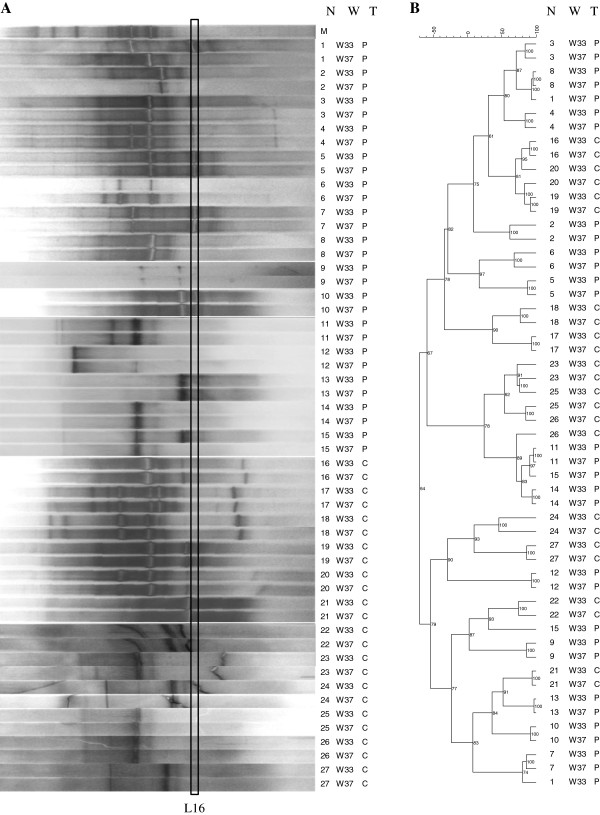
**PCR**-**DGGE analysis with *Lactobacillus***-**specific primers.** Analysis was conducted on the vaginal samples collected at 33^rd^ (W33) and 37^th^ (W37) week of gestation from 15 women supplemented with the probiotic VSL#3 [(P) N. 1–15] and 12 control women [(C) N. 16–27]. N: woman number; W: week of gestation; T: type of supplementation. (**A**) PCR-DGGE fingerprints. M, external reference marker. Band L16 corresponds to *L*. *helveticus* (GenBank accession number: AB571603) (**B**) Dendrogram of the DGGE profiles shown in panel **A**. Pearson correlation was used to calculate the similarity in DGGE profiles.

Because of the importance of lactobacilli in the establishment of a healthy vaginal environment [2], DGGE analysis with *Lactobacillus*-specific primer set (Lac1/Lac2-GC) was also carried out. This analysis allowed us to investigate the variations in lactobacilli population occurring physiologically from W33 and W37 and potentially associated with the VSL#3 intake (Figure 2). 

Richness indexes ranged from 5.7 (W33) to 5.4 (W37) for P group and from 6.3 (W33) to 6.8 (W37) for C group. Mean values of SI were 79% and 80% for P and C groups, respectively (Table 1). Only 2 women included in P group showed SIs < 50% (N. 1 and 15). Wilcoxon Signed Rank Test highlighted significant differences between DGGE profiles related to W33 and W37 for women N. 7 and 10, accounting for 13% of women included in P group. Comparing this percentage with the 33% obtained by DGGE analysis with HDA1-GC/HDA2 primer set, the probiotic intake seemed to have a more extended impact on total bacteria than lactobacilli. Notably, only for woman N. 10, significant differences were found between W33- and W37-related DGGE patterns for both HDA1-GC/HDA2 and Lac1/Lac2-GC primer sets.

The peak height analysis by Wilcoxon Signed Rank Test allowed us to identify a band, denominated L16 (Figure 2), which significantly changed after probiotic supplementation. Sequencing of the DNA extracted from this band revealed 100% homology with *L*. *helveticus* strains. The nucleotide sequence of this DGGE fragment was deposited in DDBJ Nucleotide Sequence Database under the accession number AB571603. *L*. *helveticus* was found to be a representative species within lactobacilli population since it was detected in 9 women supplemented with VSL#3 and 2 control women, corresponding to a frequency of occurrence of 40.7%. Notably, a general decrease in the intensity of *L*. *helveticus* band was observed in P group while no variations were appreciable in C group.

Cluster analysis showed that *Lactobacillus*-specific DGGE profiles related to the time points W33 and W37 were closely related for all control women and for the majority of women administered with VSL#3, except for the subjects N. 1 and 15 (Figure 2).

### Quantitative variations of vaginal bacterial populations

**Figure 3:**
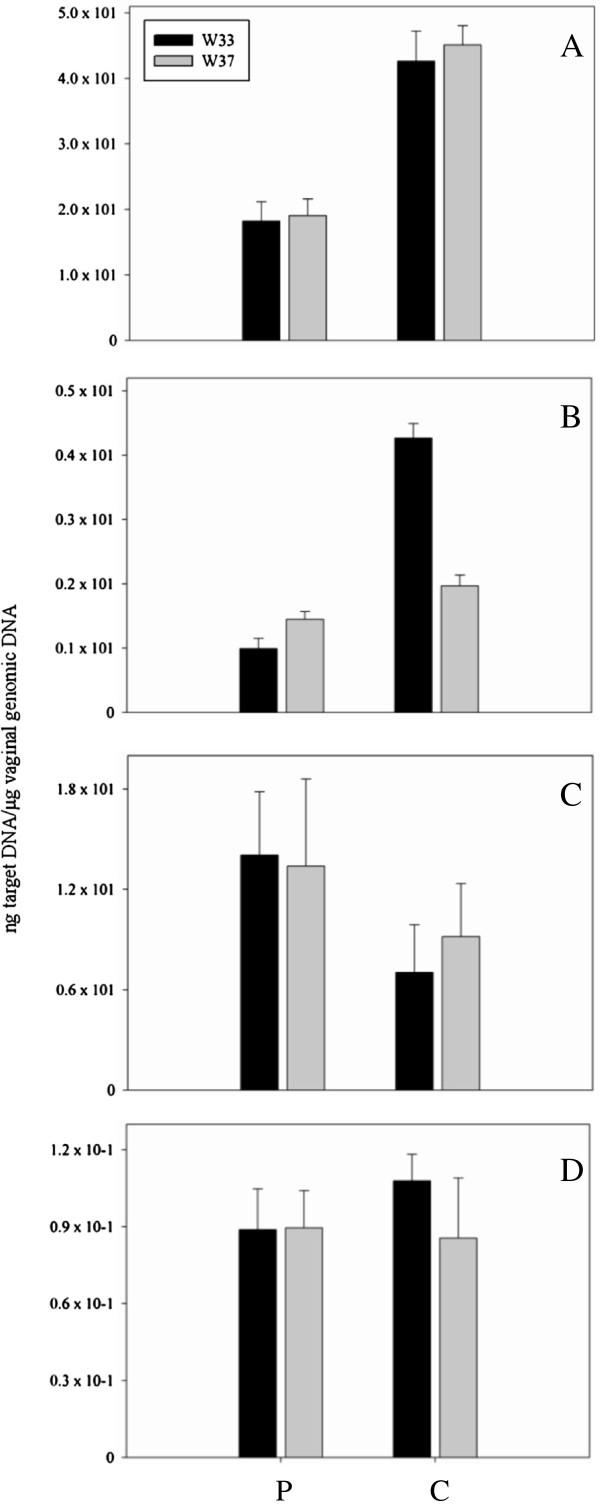
**qPCR evaluation of *Lactobacillus*(A),*Bifidobacterium*(B),*Atopobium*(C)and*Prevotella*(D).** Analysis was performed on vaginal samples collected at 33^rd^ (W33) and 37^th^ (W37) week of gestation from pregnant women supplemented (P) and not supplemented (C) with VSL#3. Data are expressed as ng of DNA of the target genus per μg of total bacterial DNA extracted from the vaginal sample. The diagrams show the mean values with error bars representing the standard deviations.

Quantitative real-time PCR (qPCR) was performed to analyze changes in concentration of *Lactobacillus*, *Bifidobacterium* and *Streptococcus thermophilus*, that were included in the probiotic VSL#3, and *Gardnerella vaginalis*, *Atopobium*, *Prevotella* and *Veillonella*, that are important BV-related genera and species [22,28]. qPCR efficiency for all assays was between 90% and 110% and correlation coefficients for genomic DNA standards were > 0.99. The sensitivity of qPCR assays was 9.1 × 10^-3^, 1.5 × 10^-4^, 3.7 × 10^-4^, 1.7 × 10^-1^, 1.4 × 10^-2^, 4.9 × 10^-4^, 3.3 × 10^-1^ ng of target DNA for *Lactobacillus*, *Bifidobacterium*, *S*. *thermophilus*, *G*. *vaginalis*, *Atopobium*, *Prevotella* and *Veillonella*, respectively. All subjects naturally harbored strains belonging to *Lactobacillus*, *Bifidobacterium*, *Atopobium* and *Prevotella*, as demonstrated by the presence of these genera in the vaginal samples collected at W33. Woman N. 9 (P group) was the only exception lacking lactobacilli at both the baseline and after one-month intake of VSL#3 (Table 2). *G*. *vaginalis* was found in two women belonging to C group (N. 18 and 20) at both time points at the concentration of 5.5 × 10^1^ ± 3.8 (N. 18: W33), 7.5 × 10^1^ ± 4.6 (N. 18: W37), 2.2 × 10^2^ ± 1.8 × 10^1^ (N. 20: W33) and 1.9 × 10^2^ ± 3.2 × 10^1^ (N. 20: W37). *S*. *thermophilus* and *Veillonella* were not detected in any pregnant woman enrolled in this study. Statistical elaboration of qPCR data related to *Lactobacillus*, *Bifidobacterium*, *Atopobium* and *Prevotella* was performed to search for significant variations of these genera associated with the going on of pregnancy or the probiotic supplementation (Figure 3). No significant changes in the amounts of these bacteria were found between W33 and W37 in both P and C groups. However, in spite of the lack of statistical relevance, a weak modulation was observed for *Bifidobacterium* and *Atopobium*. Regarding bifidobacteria (Figure 3B), a physiological tendency to decrease was observed in vaginal samples of control women at the end of the study period (mean value, W33: 4.3 ± 2.2 × 10^-1^; W37: 2.0 ± 1.7 × 10^-1^). This trend seemed to be counterbalanced in women consuming VSL#3 since amount of bifidobacteria slightly increased during the supplementation period (mean value, W33: 9.9 × 10^-1^ ± 1.6 × 10^-1^; W37: 1.4 ± 1.2 × 10^-1^). An opposite trend was observed for *Atopobium* (Figure 3C). This genus increased at W37 (mean value, 9.2 ± 3.2) compared to W33 (mean value, 7.0 ± 2.8) in C group, while it remained constant after VSL#3 supplementation (mean value, W33: 1.4 × 10^1^ ± 3.8; W37: 1.3 × 10^1^ ± 5.2). 

**Table 2:** **qPCR data of *Lactobacillus***, ***Bifidobacterium***, ***Atopobium *and *Prevotella***

		**ng of target DNA/μg vaginal genomic DNA** (**mean** ± **SD**)			
**Woman N.**	**Time point**	***Lactobacillus***	***Bifidobacterium***	***Atopobium***	***Prevotella***
Probiotic (P)					
1	W33	2.4 × 10^1^ ± 1.1	1.9 × 10^-2^ ± 7.4 × 10^-3^	3.6 ± 1.5	2.1 × 10^-2^ ± 1.0 × 10^-2^
	W37	3.0 × 10^1^ ± 3.1	3.1 × 10^-2^ ± 2.7 × 10^-4^	1.3 × 10^1^ ± 6.8	9.1 × 10^-2^ ± 1.6 × 10^-2^
2	W33	9.6 ± 8.7 × 10^-1^	3.1 × 10^-2^ ± 8.8 × 10^-3^	5.4 × 10^1^ ± 7.4	1.4 × 10^-1^ ± 4.8 × 10^-2^
	W37	5.9 × 10^-1^ ± 4.9 × 10^-2^	2.4 × 10^-2^ ± 1.2 × 10^-2^	2.4 × 10^1^ ± 1.9 × 10^1^	1.1 × 10^-1^ ± 1.1 × 10^-2^
3	W33	2.4 × 10^1^ ± 2.9	2.4 × 10^-2^ ± 4.2 × 10^-3^	1.1 × 10^1^ ± 6.0	1.1 × 10^-1^ ± 7.7 × 10^-3^
	W37	2.2 × 10^1^ ± 2.4	3.0 × 10^-2^ ± 2.4 × 10^-3^	4.0 ± 2.3	5.2 × 10^-2^ ± 8.2 × 10^-3^
4	W33	2.2 × 10^1^ ± 2.0	6.8 × 10^-2^ ± 8.3 × 10^-3^	4.7 ± 1.9	7.3 × 10^-2^ ± 2.9 × 10^-2^
	W37	1.5 × 10^1^ ± 1.4	2.1 × 10^-2^ ± 7.2 × 10^-3^	5.2 ± 2.0	4.6 × 10^-2^ ± 9.5 × 10^-3^
5	W33	2.5 × 10^1^ ± 4.5	2.1 × 10^-2^ ± 3.4 × 10^-3^	1.2 × 10^1^ ± 3.0	9.3 × 10^-2^ ± 8.3 × 10^-3^
	W37	2.2 x 10^1^ ± 4.5	1.4 x 10^-2^ ± 3.2 x 10^-3^	1.5 x 10^1^ ± 1.9	3.0 x 10^-2^ ± 1.1 x 10^-2^
6	W33	1.1 × 10^-1^ ± 3.4 × 10^-3^	7.1 × 10^-2^ ± 7.1 × 10^-3^	1.0 × 10^1^ ± 4.1	1.2 × 10^-1^ ± 1.3 × 10^-2^
	W37	2.2 ± 6.0 × 10^-1^	2.1 ± 1.7 × 10^-1^	2.4 × 10^1^ ± 1.0 × 10^1^	1.5 × 10^-1^ ± 1.2 × 10^-2^
7	W33	4.1 × 10^1^ ± 8.5	3.7 × 10^-2^ ± 5.4 × 10^-3^	2.9 × 10^1^ ± 9.2	1.2 × 10^-1^ ± 2.1 × 10^-2^
	W37	2.0 × 10^1^ ± 2.6	1.7 × 10^-2^ ± 4.4 × 10^-3^	2.6 × 10^1^ ± 7.7	1.1 × 10^-1^ ± 1.1 × 10^-3^
8	W33	1.0 × 10^1^ ± 1.7 × 10^-1^	1.3 × 10^-2^ ± 1.9 × 10^-3^	5.5 ± 1.2	4.2 × 10^-2^ ± 1.9 × 10^-2^
	W37	2.1 × 10^1^ ± 2.0	1.5 × 10^-2^ ± 2.6 × 10^-3^	1.6 × 10^1^ ± 6.6	5.1 × 10^-2^ ± 3.3 × 10^-3^
9	W33	0.0 ± 0.0	7.1 × 10^-3^ ± 2.8 × 10^-5^	1.8 × 10^1^ ± 7.1	6.7 × 10^-2^ ± 1.5 × 10^-2^
	W37	0.0 ± 0.0	1.1 × 10^1^ ± 1.0	1.5 × 10^1^ ± 6.8	2.3 × 10^-1^ ± 8.0 × 10^-2^
10	W33	6.7 ± 6.1 × 10^-1^	2.0 × 10^-2^ ± 4.8 × 10^-3^	1.4 × 10^1^ ± 4.3	8.6 × 10^-2^ ± 2.0 × 10^-2^
	W37	1.1 × 10^1^ ± 1.4	2.3 × 10^-2^ ± 1.5 × 10^-2^	1.7 × 10^1^ ± 9.7	8.0 × 10^-2^ ± 2.9 × 10^-2^
11	W33	2.7 × 10^1^ ± 1.7	2.9 x 10^-3^ ± 1.7 × 10^-3^	2.3 ± 1.8	3.2 × 10^-2^ ± 3.3 × 10^-3^
	W37	3.0 × 10^1^ ± 5.6	1.3 x 10^-2^ ± 8.5 × 10^-3^	1.3 ± 7.5 × 10^-1^	3.6 × 10^-2^ ± 1.3 × 10^-2^
12	W33	2.2 ± 5.6 × 10^-1^	1.5 × 10^1^ ± 2.3	1.4 × 10^1^ ± 2.9	2.2 × 10^-1^ ± 2.1 × 10^-2^
	W37	2.0 ± 3.1 × 10^-1^	8.7 ± 5.6 × 10^-1^	1.2 × 10^1^ ± 2.3	1.0 × 10^-1^ ± 1.8 × 10^-2^
13	W33	3.7 × 10^1^ ± 5.4	3.0 × 10^-2^ ± 4.5 × 10^-3^	7.0 ± 2.6 × 10^-1^	2.7 × 10^-2^ ± 5.0 × 10^-4^
	W37	6.6 × 10^1^ ± 5.9	1.1 × 10^-2^ ± 2.2 × 10^-3^	6.8 ± 6.6 × 10^-1^	5.7 × 10^-2^ ± 2.0 × 10^-3^
14	W33	2.2 × 10^1^ ± 8.5	1.7 × 10^-2^ ± 4.9 × 10^-3^	9.0 ± 4.4 × 10^-1^	6.7 × 10^-2^ ± 6.6 × 10^-3^
	W37	1.6 × 10^1^ ± 4.9	2.8 × 10^-2^ ± 4.7 × 10^-3^	1.1 × 10^1^ ± 1.1	1.1 × 10^-1^ ± 1.8 × 10^-3^
15	W33	2.2 × 10^1^ ± 7.1	1.4 × 10^-2^ ± 7.1 × 10^-3^	1.8 × 10^1^ ± 5.6	1.1 × 10^-1^ ± 1.4 × 10^-2^
	W37	2.8 × 10^1^ ± 3.4	4.7 × 10^-3^ ± 2.3 × 10^-3^	1.1 × 10^1^ ± 2.4 × 10^-1^	7.4 × 10^-2^ ± 2.4 × 10^-3^
Control (C)					
16	W33	5.4 × 10^1^ ± 4.0	2.1 × 10^-2^ ± 5.6 × 10^-3^	1.1 × 10^1^ ± 4.6	6.8 × 10^-2^ ± 1.1 × 10^-2^
	W37	2.0 × 10^1^ ± 1.7	2.0 × 10^-2^ ± 7.4 × 10^-3^	1.4 × 10^1^ ± 5.0	5.6 × 10^-2^ ± 5.4 × 10^-3^
17	W33	5.5 ± 5.3 × 10^-1^	6.0 ± 1.6 × 10^-1^	1.2 × 10^1^ ± 4.3	5.9 × 10^-2^ ± 2.3 × 10^-2^
	W37	1.5 × 10^1^ ± 2.9	9.3 ± 5.3 × 10^-1^	1.9 × 10^1^ ± 8.7	5.4 × 10^-2^ ± 1.0 × 10^-2^
18	W33	2.6 ± 1.6 × 10^-1^	1.8 ± 3.5 × 10^-2^	1.3 × 10^1^ ± 5.5	8.8 × 10^-2^ ± 1.7 × 10^-2^
	W37	1.2 × 10^1^ ± 2.0	2.9 ± 7.5 × 10^-2^	3.3 × 10^1^ ± 4.4	4.5 × 10^-2^ ± 2.8 × 10^-3^
19	W33	7.6 × 10^1^ ± 3.3 × 10^-1^	1.2 ± 7.9 × 10^-3^	1.3 × 10^1^ ± 3.6	1.9 × 10^-1^ ± 3.2 × 10^-3^
	W37	2.7 × 10^1^ ± 3.8	2.7 × 10^-2^ ± 4.7 × 10^-3^	8.2 ± 4.6	1.1 × 10^-1^ ± 2.6 × 10^-2^
20	W33	1.6 × 10^1^ ± 1.4	1.1 × 10^1^ ± 1.2	1.2 × 10^1^ ± 5.5	8.6 × 10^-2^ ± 1.5 × 10^-2^
	W37	1.0 × 10^1^ ± 6.4 × 10^-2^	1.1 × 10^1^ ± 1.4	1.2 × 10^1^ ± 4.7	1.1 × 10^-1^ ± 3.1 × 10^-2^
21	W33	5.6 × 10^1^ ± 8.3	1.7 × 10^-2^ ± 1.7 × 10^-3^	2.1 × 10^1^ ± 1.0 × 10^1^	1.3 × 10^-1^ ± 2.0 × 10^-2^
	W37	6.4 × 10^1^ ± 1.5	3.3 × 10^-2^ ± 8.7 × 10^-3^	2.2 × 10^1^ ± 1.0 × 10^1^	1.2 × 10^-1^ ± 2.4 × 10^-2^
22	W33	4.3 × 10^1^ ± 2.0	1.2 × 10^-1^ ± 2.8 × 10^-2^	2.3 × 10^-1^ ± 1.5 × 10^-2^	0.0 ± 0.0
	W37	6.8 × 10^1^ ± 5.1	2.7 × 10^-2^ ± 6.6 × 10^-3^	1.9 × 10^-1^ ± 2.0 × 10^-2^	0.0 ± 0.0
23	W33	2.6 × 10^1^ ± 5.6	2.3 × 10^-1^ ± 3.6 × 10^-2^	0.0 ± 0.0	0.0 ± 0.0
	W37	6.3 × 10^1^ ± 2.0	8.2 × 10^-3^ ± 1.9 × 10^-3^	1.6 × 10^-1^ ± 2.9 × 10^-2^	5.3 × 10^-1^ ± 1.8 × 10^-1^
24	W33	1.2 × 10^1^ ± 1.0	2.7 × 10^1^ ± 2.1 × 10^-1^	1.8 ± 1.5 × 10^-1^	6.8 × 10^-1^ ± 3.4 × 10^-2^
	W37	7.5 × 10^1^ ± 3.8	9.7 × 10^-3^ ± 3.7 × 10^-3^	3.7 × 10^-1^ ± 3.4 × 10^-2^	0.0 ± 0.0
25	W33	6.5 × 10^1^ ± 1.0 × 10^1^	3.0 × 10^-2^ ± 1.0 × 10^-2^	7.5 × 10^-2^ ± 7.5 × 10^-3^	0.0 ± 0.0
	W37	6.6 × 10^1^ ± 7.1	9.1 × 10^-3^ ± 5.1 × 10^-4^	2.5 × 10^-1^ ± 2.7 × 10^-2^	0.0 ± 0.0
26	W33	8.5 × 10^1^ ± 6.3	4.4 ± 9.3 × 10^-1^	3.2 × 10^-1^ ± 3.9 × 10^-2^	0.0 ± 0.0
	W37	5.4 × 10^1^ ± 4.5	2.0 × 10^-2^ ± 6.1 × 10^-4^	3.6 × 10^-1^ ± 4.2 × 10^-2^	0.0 ± 0.0
27	W33	7.0 × 10^1^ ± 1.5 × 10^1^	3.3 × 10^-2^ ± 4.7 × 10^-3^	2.8 × 10^-1^ ± 2.6 × 10^-2^	0.0 ± 0.0
	W37	6.6 × 10^1^ ± 3.6 × 10^-1^	2.1 × 10^-2^ ± 1.6 × 10^-2^	4.0 × 10^-1^ ± 3.8 × 10^-2^	0.0 ± 0.0

### Immunological profiles

The effect of the probiotic intake on the vaginal immune response was evaluated by measuring the levels of 27 cytokines, chemokines and growth factors in the vaginal samples of the pregnant women belonging to P and C groups.

**Figure 4:**
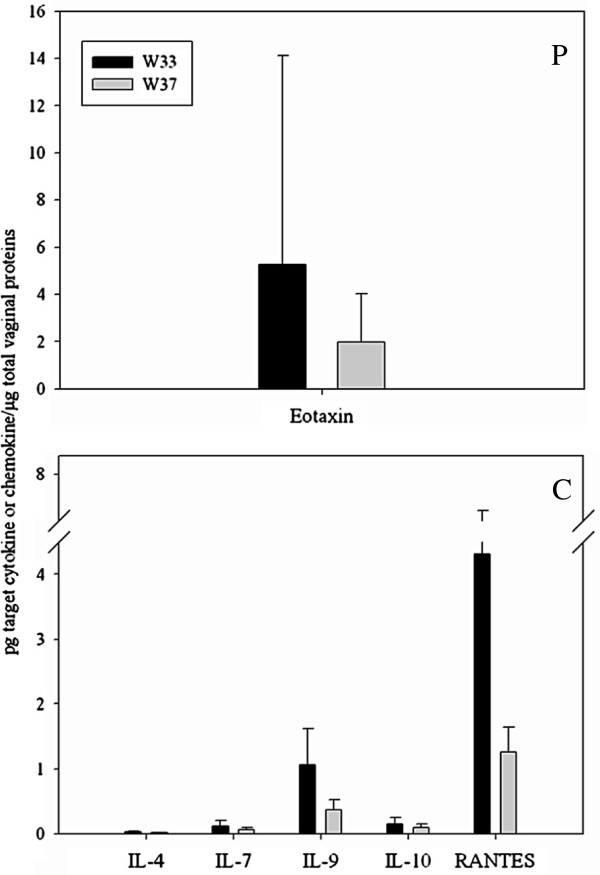
**Cytokines and chemokines whose concentration significantly changed during the study period (*P*<0**.**05).** P, probiotic group; C, control group; W33, 33^rd^ gestational week (black colour); W37, 37^th^ gestational week (grey colour). Cytokine or chemokine names are reported in x-axis. Data are expressed as pg of the target cytokine or chemokine per μg of total proteins present in the vaginal sample (y-axis). The diagrams show means with error bars representing the standard deviations.

Figure 4 shows the cytokines and chemokines whose concentration significantly changed in P and C groups during the study period (*P* < 0.05). In group C, significant reductions at W37 were found for 5 mediators, 4 cytokines [IL-4 (mean value, W33: 2.8 × 10^-2^ ± 1.5 × 10^-2^; W37: 1.3 × 10^-2^ ± 6.9 × 10^-3^), IL-7 (mean value, W33: 1.2 × 10^-1^ ± 8.6 × 10^-2^; W37: 6.1 × 10^-2^ ± 3.5 × 10^-2^), IL-9 (mean value, W33: 1.1 ± 5.6 × 10^-1^; W37: 3.7 × 10^-1^ ± 1.5 × 10^-1^) and IL-10 (mean value, W33: 1.5 × 10^-1^ ± 1.1 × 10^-1^; W37: 9.4 × 10^-2^ ± 5.4 × 10^-2^)] and 1 chemokine [RANTES (mean value, W33: 4.3 ± 2.9; W37: 1.3 ± 3.9 × 10^-1^)]. Both IL-4 and IL-10 are produced by Th2 cells and exert a regulatory role in the immune response. IL-7 and IL-9 are hematopoietic growth factors that control proliferation and homeostasis of a variety of hematopoietic cells. RANTES is a pro-inflammatory chemokine which attracts monocytes, lymphocytes, basophils and eosinophils in the inflammatory response. In P group a significant variation was registered only for the chemokine Eotaxin, which decreased after probiotic supplementation (mean value, W33: 5.3 ± 8.8; W37: 2.0 ± 2.1). Eotaxin exerts a pro-inflammatory activity by recruiting eosinophils during allergic responses.

**Figure 5:**
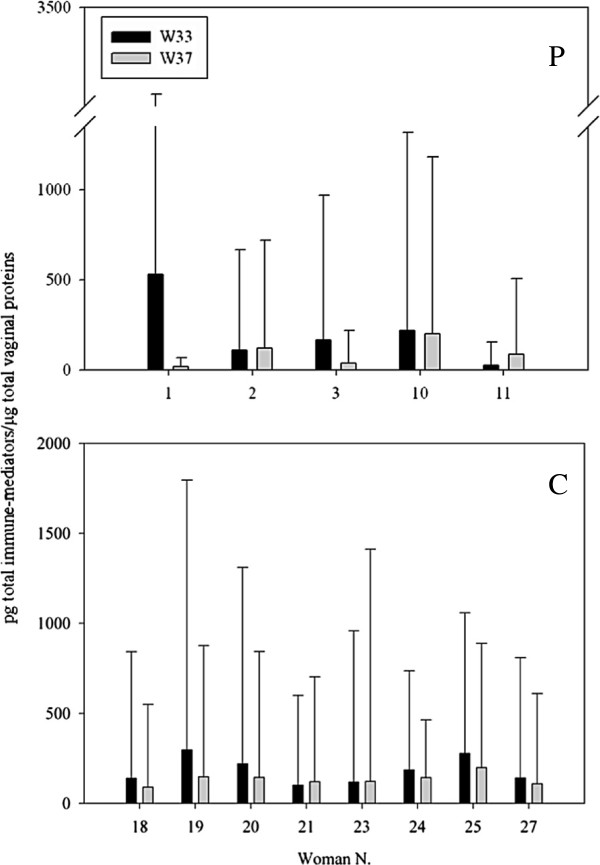
**Women registering significant variations in total levels of immune**-**mediators.** P, probiotic group; C, control group; W33, 33^rd^ gestational week (black colour); W37, 37^th^ gestational week (grey colour). Identification numbers of women registering significant variations are reported in x-axis. Data are expressed as pg of total immune-mediators per μg of total vaginal proteins (y-axis). The diagrams show means with error bars representing the standard deviations.

Figure 5 shows women, belonging to P and C groups, who registered significant variations in total levels of immune-mediators during the study period (*P* < 0.05). Significant changes were found for women N. 18, 19, 20, 21, 23, 24, 25 and 27 (8/12; 67%) of C group and women N. 1, 2, 3, 10, 11 (5/15; 33%) of P group.

## Discussion

To our knowledge, this is the first study describing the effect of a probiotic mixture, orally consumed during the last trimester of pregnancy, on the vaginal microbiota and immune response. Although several health-promoting activities of probiotics have been described in relation to the gut homeostasis [16,32], less information is available regarding the interactions between orally administered probiotic bacteria and the vaginal microbial habitat.

The first step in ascertaining the influence of the dietary supplementation with the probiotic VSL#3 on the vaginal microbiota of pregnant women was the characterization of vaginal bacterial communities by using an integrated approach based on PCR-DGGE and qPCR.

DGGE population profiling, conducted with universal primers for bacteria and *Lactobacillus*-specific primers, allowed us to investigate the variations of the predominant vaginal bacterial communities and *Lactobacillus* species occurring both physiologically in the last trimester of pregnancy and potentially associated with VSL#3 intake. The influence of the probiotic intake in modulating the predominant bacterial populations and *Lactobacillus* species could be hypothesized since significant differences between DGGE profiles at W33 and W37 were found only in women belonging to P group. Notably, the lower percentage of women belonging to P group who displayed significant differences in *Lactobacillus*-specific DGGE profiles between W33 and W37, compared to the universal bacterial DGGE patterns, suggested a major stability of lactobacilli population and a more extended impact of the probiotic VSL#3 on total bacteria than lactobacilli. However, no significant changes in single species abundances were found between W33- and W37-related universal DGGE profiles. Differently, the statistical analysis of the peak heights of the *Lactobacillus*-specific DGGE densitometric curves allowed us to identify a band, corresponding to *L*. *helveticus*, which significantly decreased after probiotic supplementation. Strains belonging to *L*. *helveticus* are used as starter cultures in the manufacturing of a variety of fermented dairy products, to modulate flavor. The presence of *L*. *helveticus* in vagina, likely due to the migration from the gut, can be related to a diet rich in yogurt and cheese. This work is not the first describing *L*. *helveticus* in vaginal samples. Stoyancheva *et al*. [33] identified this species among several *Lactobacillus* isolates from vaginal fluids of healthy Bulgarian women in childbearing age by using three different molecular techniques, amplified ribosomal DNA restriction analysis, ribotyping and PCR with species-specific primers. The decrease of *L*. *helveticus* observed in our study could be due to a competition between the *Lactobacillus* strains present in VSL#3 formula and dairy *L*. *helveticus* strains in colonizing vaginal environment.

Cluster analysis showed that universal and *Lactobacillus*-specific DGGE profiles related to the time points W33 and W37 of the control women were closely related. Also the DGGE patterns of the majority of women administered with VSL#3 grouped according to the subject and not to the time point, revealing that the inter-individual variability was higher than variability induced by the probiotic supplementation.

The hypothesis of a positive action of VSL#3 on the vaginal microbiota of pregnant women was further supported by qPCR results, which suggested a role of the probiotic product in counteracting the decrease of the health-promoting *Bifidobacterium* genus and the increase of the BV-related *Atopobium* genus, that occurred in control women during late pregnancy. Notably, group B *Streptococcus*, which was found in two women (N.1 and 10) before the probiotic intake, was no longer found after the dietary supplementation (data not shown).

The second step of the present research was the investigation of the vaginal immunological profiles of the pregnant women in order to search for correlations between the VSL#3 intake and changes in vaginal immune response. Pregnancy has been referred to as a state of relative immune compromise. This notion has been related to both demonstration of depression of certain aspects of cell-mediated immunity and clinical observations of an increased severity of numerous infectious conditions in pregnant women [7]. On the other hand, preterm cervical ripening can be likened to an inflammatory process with cytokines as important mediators [34].

Bioplex immunoassay was used in the present work to measure levels of 27 cytokines, chemokines and growth factors in the vaginal samples of the pregnant women belonging to P and C groups. In group C a significant reduction at W37 was found for IL-4, IL-7, IL-9, IL-10 and RANTES. IL-4 is a key regulator in humoral and adaptive immunity. It has many biological roles, including the stimulation of activated B-cells and T-cell proliferation, and the differentiation of CD4+ T-cells into Th2 cells. A regulatory role is also exerted by IL-10. In relation to pregnancy, IL-10 decreases the production of pro-inflammatory cytokines, such as IL-8, IL-6, TNFα, IL-1β and prostaglandin E_2_ in lipopolysaccharide-stimulated fetal membranes [35,36]. Both IL-4 and IL-10 are produced by Th2 cells. IL-7 and IL-9 are hematopoietic growth factors that act as regulators of cell survival, proliferation and homeostasis of a variety of hematopoietic cells. RANTES is a potent and versatile chemokine, capable of attracting monocytes, lymphocytes, basophils and eosinophils. This cytokine has been implicated in the regulation of the inflammatory response and recruitment of macrophages to the implantation site in early pregnancy [37]. However, no variations in RANTES levels have been associated with preterm cervical ripening and labor [34]. Immunological profiles related to women belonging to C group indicated that some fluctuations in vaginal immune-modulators occurred physiologically during the last trimester of pregnancy. In particular, it is noteworthy the decrease of IL-10 and IL-4, important regulatory cytokines controlling the inflammatory reaction responsible for uterine contractions and cervical ripening at the labor time [12]. In P group a significant variation was registered only for the chemokine Eotaxin, which decreased after probiotic supplementation. Eotaxin selectively recruits eosinophils, and for this reason is implicated in allergic responses [38]. By comparing the data related to the two study groups, the following hypotheses could be formulated regarding the possible impact of the probiotic intake on cytokine secretion during late pregnancy: (i) probiotics counteracted the decrease of anti-inflammatory cytokine levels occurring in C group; (ii) probiotics induced the decrease of a pro-inflammatory cytokine in P group, showing a global anti-inflammatory effect on the vaginal immunity. In addition, a stabilization effect on the vaginal immunity during late pregnancy could be attributed to the probiotic intake, since the percentage of women with modified amounts of immune-mediators decreased from 67% to 31% in relation to the dietary supplementation.

## Conclusion

The impact of the oral intake of the probiotic VSL#3 on the vaginal microbiota and immune response of pregnant women was investigated by molecular fingerprinting techniques (PCR-DGGE and qPCR) and Luminex® immunoassay. The major findings of this study are the following: (i) VSL#3 intake seems to be associated with a modulation of the predominant vaginal bacterial communities; (ii) VSL#3 modulation of *Lactobacillus* population appears to be related to variations of *L*. *helveticus* species; (iii) a potential role of the probiotic product in counteracting the physiological decrease of *Bifidobacterium* and increase of *Atopobium* could be hypothesized; (iv) the probiotic supplementation can be associated with a global anti-inflammatory effect on the vaginal immunity, with potential implications in preventing preterm birth.

## Methods

### Study design and sample collection

A pilot, not randomized, controlled and perspective study was conducted. The study protocol was approved by the ethical committee of the University of Bari, Italy. Written informed consent was obtained from all the participants in the study. A total of 27 healthy pregnant women (21 to 42 years of age; mean, 32) who had no symptoms of vaginal or urinary tract infection were included in the present study (Table 3). None of the subjects had received oral or local antimicrobial therapy within the previous 2 weeks. The recruited subjects were divided into 2 groups: (i) probiotic group [P (n=15)]; (ii) control group [C (n=12)] on the basis of their availability to consume the probiotic product. Women of the P group consumed 1 sachet once/day of VSL#3 (VSL Pharmaceuticals, Inc.,Towson, MD, USA) for 4 weeks from the 33^rd^ (W33) to the 37^th^ (W37) week of gestation. Women of the C group did not receive any dietary supplementation. VSL#3 sachet contains 900 billion viable lyophilized bacteria consisting of 4 strains of *Lactobacillus* (*L*. *paracasei*, *L*. *plantarum*, *L*. *acidophilus*, *L*. *delbrueckii* subsp. *bulgaricus*), 3 strains of *Bifidobacterium* (*B*. *longum*, *B*. *breve*, *B*. *infantis*) and 1 strain of *Streptococcus thermophilus*. Mid-vaginal swabs were collected from women of both P and C groups at the time points W33 and W37. Samples were placed in 1 ml of sterile saline and stored immediately at −80°C until use.

**Table 3:** Characterization of the subjects included in the study groups

**Woman N**	**Age**	**Type of delivery**^**1**^	**Gestational age at birth**
Probiotic (n = 15)			
1	31	SD	39 week + 6 days
2	32	CD	40 week + 3 days
3	39	SD	40 week + 1 day
4	31	SD	40 week + 2 days
5	33	SD	40 week + 3 days
6	30	SD	39 week
7	33	SD	41 week + 3 days
8	34	CD	39 week
9	36	CD	38 week + 4 days
10	38	SD	38 week + 5 days
11	42	SD	39 week + 4 days
12	30	SD	39 week
13	29	SD	40 week + 2 days
14	33	CD	39 week + 2 days
15	25	SD	40 week + 1 day
Control (n = 12)			
16	28	SD	40 week + 6 days
17	33	SD	39 week + 3 days
18	33	CD	37 week + 4 days
19	32	CD	41 week + 3 days
20	34	SD	40 week
21	21	SD	39 week + 5 days
22	30	SD	38 week + 6 days
23	30	SD	40 week + 2 days
24	34	CD	39 week + 6 days
25	38	CD	41 week + 1 days
26	38	CD	38 week + 5 days
27	30	SD	40 week + 2 days

^1^ SD: spontaneous delivery; CD: caesarean delivery.

The individual characteristics (age, type of delivery and gestational age at birth) of women enrolled in the present study are reported in Table 3. Gestational age was determined by utilizing the last menstrual period and earliest ultrasound.

### DNA extraction from vaginal samples

Frozen vaginal swabs were thawed, mixed by vortex shaker for 1 min and then removed from the liquid. The liquid was centrifuged at 10,000 × g for 15 min, and the pellet was washed 3 times in saline at 40°C. The pellet was resuspended in 180 μl of enzymatic lysis buffer (20 mM Tris–HCl, pH 8, 2 mM EDTA, 1.2% Triton X-100, 20 mg/ml lysozyme) and incubated at 37°C for 30 min. Glass beads (200 mg) were added and the sample was mixed by vortexing for 1 min. Total DNA was extracted by using the DNeasy Blood & Tissue Kit (Qiagen, Hilden, Germany) following the protocol “Pretreatment for Gram-positive bacteria”. A slight modification was introduced: a centrifugation step (8000 × g for 5 min) was carried out after incubation with proteinase K to remove glass beads. DNA amounts were quantified by using NanoDrop 1000 (Thermo Scientific, Wilmington, DE).

### PCR-DGGE and cluster analysis

Amplification reactions were performed in a Biometra Thermal Cycler T Gradient (Biometra, Göttingen, Germany). GoTaq Flexi DNA Polymerase (Promega, Madison, WI) was used as thermostable DNA polymerase. The reaction mixture contained 0.5 μM of each primer, 200 μM of each dNTP, 2 mM MgCl_2_ solution, 1.25 U of GoTaq Flexi DNA Polymerase, 5 μl of Green GoTaq Flexi buffer 5X, and 2 μl of the bacterial DNA template (30–40 ng) in a final volume of 25 μl. The universal primers HDA1-GCclamp and HDA2 for bacteria [39] were used to amplify a conserved region within the 16S rRNA gene. The thermocycle program consisted of the following time and temperature profile: 95°C for 5 min; 30 cycles of 95°C for 30 s, 56°C for 30 s, 72°C for 60 s; and 72°C for 8 min. The *Lactobacillus* genus-specific primers Lac1 and Lac2-GCclamp [40] were used to amplify a specific region of the 16S rRNA gene of lactobacilli. The amplification program was 95°C for 5 min; 35 cycles of 95°C for 30 s, 61°C for 30 s, 72°C for 60 s; and 72°C for 8 min. A volume of 8 μl of PCR samples was loaded on DGGE gels, containing 30-50% and 25-55% gradients of urea and formamide for universal bacteria and lactobacilli, respectively. DGGE analysis was performed by using the D-Code Universal Mutation System Apparatus (Bio-Rad, Los Angeles, CA), as previously described [22]. Following electrophoresis, gels were silver stained [41] and scanned using a Molecular Imager Gel Doc XR System (Bio-Rad). DGGE gel images were analyzed using the FPQuest software version 4.5 (Bio-Rad). In order to compensate for gel-to-gel differences and external distortion to electrophoresis, the DGGE patterns were aligned and normalized using an external reference marker. The marker for the DGGE analysis with the universal primers for bacteria contained PCR amplicons from *Bacteroides*, *Coriobacterium*, *Enterococcus faecalis*, *Bifidobacterium bifidum*, *Lactobacillus casei*, *Acidaminococcus fermentas* and *Atopobium*. The marker for the DGGE analysis with *Lactobacillus*-specific primers contained PCR amplicons from *L*. *plantarum*, *L*. *paracasei*, *L*. *brevis*, *L*. *gasseri*, *L*. *acidophilus* and *L*. *delbrueckii* subsp. *bulgaricus*. After normalization, bands were defined for each sample using the appropriate densitometric curve. The similarity in the profiles was calculated on the basis of the Pearson correlation coefficient with the Ward clustering algorithm. Cluster analysis of the DGGE patterns was performed using the FPQuest software.

### Sequencing of DGGE fragment

The DNA fragment of interest was excised from the denaturing gel with a sterile scalpel, washed once in 1X PCR buffer, and incubated in 20 μl of the same buffer overnight at 4°C. Two μl of the buffer solution were used as a template for PCR reaction. Reamplification of the 16S rRNA region was conducted as described above by employing the primers Lac1 and Lac2 (without the GC-clamp). The re-amplified fragment was purified using the Wizard SV Gel and PCR Clean-up system (Promega), and then subjected to automated sequence analysis of both DNA strands with Lac1 and Lac2. BigDye terminators (ABI-PerkinElmer, Foster City, CA) were used with a 377 sequencer (ABI). Sequence identity was determined by comparison with the rRNA gene sequences deposited in GenBank database using BLAST algorithm (http://www.ncbi.nlm.nih.gov/BLAST).

### Quantitative real-time PCR

Quantitative PCR was performed in a LightCycler instrument (Roche, Mannheim, Germany) and SYBR Green I fluorophore was used to correlate the amount of PCR product with the fluorescence signal. Each DNA sample was amplified with different genus- or species-specific primer sets targeted to 16S rRNA gene or 16S-23S rRNA spacer region: Bact-0011f/Lab-0677r [42] for *Lactobacillus*, Bif164/Bif662 [43] for *Bifidobacterium*, Th1/Th2 [44] for *Streptococcus thermophilus*, F-GV1/R-GV3 [45] for *Gardnerella vaginalis*, c-Atopo-f/c-Atopo-r [46] for *Atopobium*, g-Prevo-f/g-Prevo-r [47] for *Prevotella*, VeilloF/VeilloR [48] for *Veillonella*. Amplifications were carried out in a final volume of 20 μl containing 0.5 μM of each primer, 4 μl of LightCycler-FastStart DNA Master SYBR Green I (Roche) and either 2 μl of template or water (no-template control).

The thermal cycling conditions were as follows: an initial denaturation step at 95°C for 10 min followed by 30 (*Lactobacillus*, *Atopobium*, *G*. *vaginalis* and *Veillonella*), 35 (*Prevotella*) or 40 (*Bifidobacterium*, *S*. *thermophilus*) cycles of denaturation at 95°C for 15 s; primer annealing at 63°C (*Lactobacillus*, *S*. *thermophilus*), 62°C (*Veillonella*), or 60°C (*Bifidobacterium*, *Atopobium*, *Prevotella*, *G*. *vaginalis* ) for 20 s; extension at 72°C for 45 s (*Lactobacillus*, *Atopobium*, *Prevotella*, *G*. *vaginalis*, *Veillonella*), 30 s (*Bifidobacterium*), or 15 s (*S*. *thermophilus*) and a fluorescence acquisition step at 85°C (*Lactobacillus*, *Atopobium*, *G*. *vaginalis*, *Veillonella*, *S*. *thermophilus*), 87°C (*Prevotella*) or 90°C (*Bifidobacterium*) for 5 s. DNAs extracted from *L*. *acidophilus* NCFM, *B*. *longum* NCC2705, *G*. *vaginalis* ATCC 14018, *Prevotella bivia* ATCC 29303, *Veillonella parvula* ATCC 10790, *Atopobium vaginae* ATCC BAA-55 and *S*. *thermophilus* ATCC 19258 were used as standards for PCR quantification. DNAs extracted from vaginal samples were amplified in triplicate for each primer set and the mean value was used for statistical analysis. Data were expressed as ng of DNA of the targeted genus or species per μg of total DNA extracted from the vaginal sample.

### Bioplex immunoassay

Cytokine levels were determined using a multiplexed bead immunoassay. Prior to assay, vaginal samples were concentrated 10 times with Microcon spin devices (YM3, Millipore Corporation, Billerica, MA) and subsequently resuspended in Bio-Plex Assay Buffer. The levels of 27 immune-mediators, 15 cytokines (IL-1β, IL-1ra, IL-2, IL-4, IL-5, IL-6, IL-7, IL-9, IL-10, IL-12(p70), IL-13, IL-15, IL-17, IFN-γ, TNFα), 7 chemokines (MCP-1, MIP-1α, MIP-1β, RANTES, Eotaxin, IL-8, IP-10) and 5 growth factors (PDGF-BB, FGF basic, G-CSF, GM-CSF, VEGF), were measured using the human ultrasensitive cytokine 27-plex antibody bead kit (Bio-Rad). Assays were performed in 96-well filter plates, as previously described [49]. Briefly, the filter plate was prewetted with washing buffer (Bio-Rad) and the solution was aspirated from the wells using a vacuum manifold (Millipore Corporation). Microsphere beads coated with monoclonal antibodies against the different target analytes were added to the wells. Samples and standards were pipetted into the wells and incubated for 30 min with the beads. Wells were washed using a vacuum manifold (Millipore Corporation) and biotinylated secondary antibodies were added. After incubation for 30 min, beads were washed then incubated for 10 min with streptavidin-PE conjugated to the fluorescent protein, R-phycoerythrin (streptavidin/R-phycoerythrin). After washing to remove the unbound streptavidin/R-phycoerythrin, the beads (a minimum of 100 per analyte) were analyzed in the Luminex 200 instrument (MiraiBio, Alameda, CA). The Luminex 200 monitors the spectral properties of the beads to distinguish the different analytes, while simultaneously measuring the amount of fluorescence associated with R-phycoerythrin, reported as median fluorescence intensity. The concentration of the samples was estimated from the standard curve using a fifth-order polynomial equation and expressed as pg/ml after adjusting for the dilution factor (Bio-Plex Manager software version 5.0). Samples below the detection limit of the assay were recorded as zero, while samples above the upper limit of quantification of the standard curves were assigned the highest value of the curve. The intra-assay CV including ultrafiltration and immunoassay averaged 19%. Concentrations of cytokines, chemokines and growth factors were then converted in pg of the target molecule per μg of total proteins present in the vaginal sample.

### Statistical analysis

Statistical analysis was performed using SigmaStat (Systat Software, Point Richmond, CA). For each subject, variations of the DGGE profiles related to the time points W33 and W37 were analyzed by Pearson correlation. Significant differences in the intensity of each DGGE band among all vaginal samples and in the amounts of the bacterial genera and species determined by qPCR were searched by using Wilcoxon Signed Rank Test. This test was also used to analyze differences in cytokines, chemokines and growth factors. A *P* value below 0.05 was considered statistically significant.

## Competing interests

VSL Pharmaceuticals, Inc. is financing the article-processing charge. The authors declare that they have no other competing interests.

## Authors’ contributions

BV performed the study design, analysis and interpretation of the data and the writing of the paper. FC, MC and ST performed DGGE and qPCR experiments and statistical analysis of the data. MEB and TC enrolled the subjects and collected the vaginal samples. ES and MCV carried out the Bioplex immunoassay. PB supervised the study. All authors read and approved the manuscript.

## References

[B1] Lidbeck A Nord CE Lactobacilli and the normal human anaerobic microflora Clin Infect Dis 1993 16 Suppl 4 181 187 8324115 10.1093/clinids/16.supplement_4.s181

[B2] Donati L Di Vico A Nucci M Quagliozzi L Spagnuolo T Labianca A Bracaglia M Ianniello F Caruso A Paradisi G Vaginal microbial flora and outcome of pregnancy Arch Gynecol Obstet 2010 281 589 600 10.1007/s00404-009-1318-3 19967381

[B3] Mattison DR Damus K Fiore E Petrini J Alter C Preterm delivery: a public health perspective Paediatr Perinat Epidemiol 2001 15 Suppl 2 7 16 11520396 10.1046/j.1365-3016.2001.00004.x

[B4] Goldenberg RL Culhane JF Iams JD Romero R Epidemiology and causes of preterm birth Lancet 2008 371 75 84 10.1016/S0140-6736(08)60074-4 18177778 PMC7134569

[B5] Hillier SL Nugent RP Eschenbach DA Krohn MA Gibbs RS Martin DH Cotch MF Edelman R Pastorek JG Rao AV McNellis D Regan JA Carey JC Klebanoff MA Association between bacterial vaginosis and preterm delivery of a low-birth-weight infant. The vaginal infections and prematurity study group N Engl J Med 1995 333 1737 1742 10.1056/NEJM199512283332604 7491137

[B6] McGregor JA French JI Bacterial vaginosis in pregnancy Obstet Gynecol Surv 2000 55 5 Suppl 1 1 19 10804540 10.1097/00006254-200005001-00001

[B7] Beigi RH Yudin MH Cosentino L Meyn LA Hillier SL Cytokines, pregnancy, and bacterial vaginosis: comparison of levels of cervical cytokines in pregnant and nonpregnant women with bacterial vaginosis J Infect Dis 2007 196 1355 1360 10.1086/521628 17922400

[B8] Mattsby-Baltzer I Platz-Christensen JJ Hosseini N Rosén P IL-1beta, IL-6, TNFalpha, fetal fibronectin, and endotoxin in the lower genital tract of pregnant women with bacterial vaginosis Acta Obstet Gynecol Scand 1998 77 701 706 10.1080/j.1600-0412.1998.770701.x 9740515

[B9] Norwitz ER Robinson JN Challis JR The control of labor N Engl J Med 1999 341 660 666 10.1056/NEJM199908263410906 10460818

[B10] Challis JR Lockwood CJ Myatt L Norman JE Strauss JF Petraglia F Inflammation and pregnancy Reprod Sci 2009 16 206 215 10.1177/1933719108329095 19208789

[B11] Houben ML Nikkels PG van Bleek GM Visser GH Rovers MM Kessel H de Waal WJ Schuijff L Evers A Kimpen JL Bont L The association between intrauterine inflammation and spontaneous vaginal delivery at term: a cross-sectional study PLoS One 2009 4 e6572 10.1371/journal.pone.0006572 19668329 PMC2718580

[B12] Dubicke A Fransson E Centini G Andersson E Byström B Malmström A Petraglia F Sverremark-Ekström E Ekman-Ordeberg G Pro-inflammatory and anti-inflammatory cytokines in human preterm and term cervical ripening J Reprod Immunol 2010 84 176 185 10.1016/j.jri.2009.12.004 20096464

[B13] FAO/WHO Guidelines for the evaluation of probiotics in food 2002 Food and Agriculture Organization of United Nations and World Health Organization Working Group report, London, Ontario

[B14] Reid G Bocking A The potential for probiotics to prevent bacterial vaginosis and preterm labor Am J Obstet Gynecol 2003 189 1202 1208 10.1067/S0002-9378(03)00495-2 14586379

[B15] Reid G Charbonneau D Erb J Kochanowski B Beuerman D Poehner R Bruce AW Oral use of Lactobacillus rhamnosus GR-1 and L. fermentum RC-14 significantly alters vaginal flora: randomized, placebo-controlled trial in 64 healthy women FEMS Immunol Med Microbiol 2003 35 131 134 10.1016/S0928-8244(02)00465-0 12628548

[B16] Reid G Anukam K James VI van der Mei HC Heineman C Busscher HJ Bruce AW Oral probiotics for maternal and newborn health J Clin Gastroenterol 2005 39 353 354 10.1097/01.mcg.0000159268.58480.02 15815200

[B17] Rautava S Kalliomäki M Isolauri E Probiotics during pregnancy and breast-feeding might confer immunomodulatory protection against atopic disease in the infant J Allergy Clin Immunol 2002 109 119 121 10.1067/mai.2002.120273 11799376

[B18] Huurre A Laitinen K Rautava S Korkeamäki M Isolauri E Impact of maternal atopy and probiotic supplementation during pregnancy on infant sensitization: a double-blind placebo-controlled study Clin Exp Allergy 2008 38 1342 1348 10.1111/j.1365-2222.2008.03008.x 18477013

[B19] Zhou X Bent SJ Schneider MG Davis CC Islam MR Forney LJ Characterization of vaginal microbial communities in adult healthy women using cultivation-independent methods Microbiology 2004 150 2565 2573 10.1099/mic.0.26905-0 15289553

[B20] Hyman RW Fukushima M Diamond L Kumm J Giudice LC Davis RW Microbes on the human vaginal epithelium Proc Natl Acad Sci U S A 2005 102 7952 7957 10.1073/pnas.0503236102 15911771 PMC1142396

[B21] Sundquist A Bigdeli S Jalili R Druzin ML Waller S Pullen KM El-Sayed YY Taslimi MM Batzoglou S Ronaghi M Bacterial flora-typing with targeted, chip-based Pyrosequencing BMC Microbiol 2007 7 108 10.1186/1471-2180-7-108 18047683 PMC2244631

[B22] Vitali B Pugliese C Biagi E Candela M Turroni S Bellen G Donders GG Brigidi P Dynamics of vaginal bacterial communities in women developing bacterial vaginosis, candidiasis, or no infection, analyzed by PCR-denaturing gradient gel electrophoresis and real-time PCR Appl Environ Microbiol 2007 73 5731 5741 10.1128/AEM.01251-07 17644631 PMC2074899

[B23] Oakley BB Fiedler TL Marrazzo JM Fredricks DN Diversity of human vaginal bacterial communities and associations with clinically defined bacterial vaginosis Appl Environ Microbiol 2008 74 4898 4909 10.1128/AEM.02884-07 18487399 PMC2519371

[B24] Kim TK Thomas SM Ho M Sharma S Reich CI Frank JA Yeater KM Biggs DR Nakamura N Stumpf R Leigh SR Tapping RI Blanke SR Slauch JM Gaskins HR Weisbaum JS Olsen GJ Hoyer LL Wilson BA Heterogeneity of vaginal microbial communities within individuals J Clin Microbiol 2009 47 1181 1189 10.1128/JCM.00854-08 19158255 PMC2668325

[B25] Burton JP Cadieux PA Reid G Improved understanding of the bacterial vaginal microbiota of women before and after probiotic instillation Appl Environ Microbiol 2003 69 97 101 10.1128/AEM.69.1.97-101.2003 12513982 PMC152440

[B26] Devillard E Burton JP Reid G Complexity of vaginal microflora as analyzed by PCR denaturing gradient gel electrophoresis in a patient with recurrent bacterial vaginosis Infect Dis Obstet Gynecol 2005 13 25 31 10.1155/2005/607474 16040324 PMC1784553

[B27] De Backer E Verhelst R Verstraelen H Alqumber MA Burton JP Tagg JR Temmerman M Vaneechoutte M Quantitative determination by real-time PCR of four vaginal * Lactobacillus * species. Gardnerella vaginalis and Atopobium vaginae indicates an inverse relationship between L. gasseri and L. iners BMC Microbiol 2007 7 115 10.1186/1471-2180-7-115 18093311 PMC2233628

[B28] Biagi E Vitali B Pugliese C Candela M Donders GG Brigidi P Quantitative variations in the vaginal bacterial population associated with asymptomatic infections: a real-time polymerase chain reaction study Eur J Clin Microbiol Infect Dis 2009 28 281 285 10.1007/s10096-008-0617-0 18762999

[B29] El Aila NA Tency I Claeys G Verstraelen H Saerens B Santiago GL De Backer E Cools P Temmerman M Verhelst R Vaneechoutte M Identification and genotyping of bacteria from paired vaginal and rectal samples from pregnant women indicates similarity between vaginal and rectal microflora BMC Infect Dis 2009 9 167 10.1186/1471-2334-9-167 19828036 PMC2770471

[B30] Guandalini S Magazzù G Chiaro A La Balestra V Di Nardo G Gopalan S Sibal A Romano C Canani RB Lionetti P Setty M VSL#3 improves symptoms in children with irritable bowel syndrome: a multicenter, randomized, placebo-controlled, double-blind, crossover study J Pediatr Gastroenterol Nutr 2010 51 24 34 10.1097/MPG.0b013e3181ca4d95 20453678

[B31] Brigidi P Vitali B Swennen E Altomare L Rossi M Matteuzzi D Specific detection of * Bifidobacterium * strains in a pharmaceutical probiotic product and in human feces by polymerase chain reaction Syst Appl Microbiol 2000 23 391 399 10.1016/S0723-2020(00)80070-3 11108019

[B32] Pagnini C Saeed R Bamias G Arseneau KO Pizarro TT Cominelli F Probiotics promote gut health through stimulation of epithelial innate immunity PNAS 2010 107 454 459 10.1073/pnas.0910307107 20018654 PMC2806692

[B33] Stoyancheva GD Danova ST Boudakov IY Molecular identification of vaginal lactobacilli isolated from Bulgarian women Antonie Van Leeuwenhoek 2006 90 201 210 10.1007/s10482-006-9072-z 16871423

[B34] Törnblom SA Klimaviciute A Byström B Chromek M Brauner A Ekman-Ordeberg G Non-infected preterm parturition is related to increased concentrations of IL-6, IL-8 and MCP-1 in human cervix Reprod Biol Endocrinol 2005 3 39 10.1186/1477-7827-3-39 16122384 PMC1201172

[B35] Fortunato SJ Menon R Lombardi SJ Interleukin-10 and transforming growth factor-beta inhibit amniochorion tumor necrosis factor-alpha production by contrasting mechanisms of action: therapeutic implications in prematurity Am J Obstet Gynecol 1997 177 803 809 10.1016/S0002-9378(97)70272-2 9369823

[B36] Brown NL Alvi SA Elder MG Bennett PR Sullivan MH The regulation of prostaglandin output from term intact fetal membranes by anti-inflammatory cytokines Immunology 2000 99 124 133 10.1046/j.1365-2567.2000.00942.x 10651950 PMC2327135

[B37] Athayde N Romero R Maymon E Gomez R Pacora P Araneda H Yoon BH A role for the novel cytokine RANTES in pregnancy and parturition Am J Obstet Gynecol 1999 181 989 994 10.1016/S0002-9378(99)70337-6 10521766

[B38] Garcia-Zepeda EA Rothenberg ME Ownbey RT Celestin J Leder P Luster AD Human eotaxin is a specific chemoattractant for eosinophil cells and provides a new mechanism to explain tissue eosinophilia Nat Med 1996 2 449 456 10.1038/nm0496-449 8597956

[B39] Walter J Tannock GW Tilsala-Timisjarvi A Rodtong S Loach DM Munro K Alatossava T Detection and identification of gastrointestinal Lactobacillus species by using denaturing gradient gel electrophoresis and species-specific PCR primers Appl Environ Microbiol 2000 66 297 303 10.1128/AEM.66.1.297-303.2000 10618239 PMC91821

[B40] Walter J Hertel C Tannock GW Lis CM Munro K Hammes WP Detection of * Lactobacillus, Pediococcus, Leuconostoc, * and * Weissella * species in human feces by using group-specific PCR primers and denaturing gradient gel electrophoresis Appl Environ Microbiol 2001 67 2578 2585 10.1128/AEM.67.6.2578-2585.2001 11375166 PMC92910

[B41] Bassam BJ Caetano-Anollés G Gresshoff PM Fast and sensitive silver staining of DNA in polyacrylamide gels Anal Biochem 1991 196 80 83 10.1016/0003-2697(91)90120-I 1716076

[B42] Heilig HGHJ Zoetendal EG Vaughan EE Marteau P Akkermans AD de Vos WM Molecular diversity of * Lactobacillus * spp. and other lactic acid bacteria in the human intestine as determined by specific amplification of 16S ribosomal DNA Appl Environ Microbiol 2002 68 114 123 10.1128/AEM.68.1.114-123.2002 11772617 PMC126540

[B43] Kok RG de Waal A Schut F Welling GW Weenk G Hellingwerf KJ Specific detection and analysis of a probiotic * Bifidobacterium * strain in infant feces Appl Environ Microbiol 1996 62 3668 3672 8837422 10.1128/aem.62.10.3668-3672.1996 PMC168175

[B44] Tilsala-Timisjärvi A Alatossava T Development of oligonucleotide primers from the 16S-23S rRNA intergenic sequences for identifying different dairy and probiotic lactic acid bacteria by PCR Int J Food Microbiol 1997 35 49 56 10.1016/S0168-1605(97)88066-X 9081225

[B45] Zariffard MR Saifuddin M Sha BE Spear GT Detection of bacterial vaginosis-related organisms by real-time PCR for Lactobacilli, * Gardnerella vaginalis * and * Mycoplasma hominis *FEMS Immunol Med Microbiol 2002 34 277 281 10.1111/j.1574-695X.2002.tb00634.x 12443827

[B46] Matsuki T Watanabe K Fujimoto J Takada T Tanaka R Use of 16S rRNA gene-targeted group-specific primers for real-time PCR analysis of predominant bacteria in human feces Appl Environ Microbiol 2004 70 7220 7228 10.1128/AEM.70.12.7220-7228.2004 15574920 PMC535136

[B47] Matsuki T Watanabe K Fujimoto J Miyamoto Y Takada T Matsumoto K Oyaizu H Tanaka R Development of 16S rRNA-gene-targeted group-specific primers for the detection and identification of predominant bacteria in human feces Appl Environ Microbiol 2002 68 5445 5451 10.1128/AEM.68.11.5445-5451.2002 12406736 PMC129894

[B48] Rinttilä T Kassinen A Malinen E Krogius L Palva A Development of an extensive set of 16S rDNA-targeted primers for quantification of pathogenic and indigenous bacteria in faecal samples by real-time PCR J Appl Microbiol 2004 97 1166 1177 10.1111/j.1365-2672.2004.02409.x 15546407

[B49] Vignali DA Multiplexed particle-based flow cytometric assays J Immunol Methods 2000 243 243 255 10.1016/S0022-1759(00)00238-6 10986418

